# Outcome of acute myeloid leukaemia in Nigeria: clinician’s perspective

**DOI:** 10.3332/ecancer.2021.1239

**Published:** 2021-05-25

**Authors:** Ann Abiola Ogbenna, Olufemi Abiola Oyedeji, Christiana Oluwakemi Famuyiwa, Babajide Ayodeji Sopekan, Obadiah Dapus Damulak, Esere Bernice Akpatason, Gbenga Olorunfemi, Kehinde Adekola

**Affiliations:** 1 Department of Haematology and Blood Transfusion, College of Medicine, University of Lagos/Lagos University Teaching Hospital, Idi-Araba, Lagos, PMB 12003, Nigeria; 2 Department of Haematology and Blood Transfusion, College of Medicine, University of Lagos, Lagos, Nigeria; 3 Department of Haematology and Blood Transfusion, General Hospital Gbagada, Lagos, Nigeria; 4 Department of Community Medicine and Primary Care, College of Medicine, University of Lagos; 5 Department of Haematology and Blood Transfusion, Faculty of Clinical Sciences, College of Health Sciences, University of Jos, Nigeria; 6 Department of Haematology, National Orthopaedic Hospital, Igbobi-Lagos, Nigeria; 7 Division of Epidemiology and Biostatistics, School of Public Health, University of Witwatersrand, Johannesburg, South Africa; 8 Hem-Onc Stem Cell Transplant Unit, Division of Hematology-Oncology, Northwestern Memorial Hospital, Northwestern University, Feinberg School of Medicine, 676 North St. Clair Street, Suite 850, IL 60611, Chicago, USA; a https://orcid.org/0000-0003-1441-2308; b https://orcid.org/0000-0001-6634-8550

**Keywords:** clinical outcome in AML, clinician perspective, cytogenetic and molecular monitoring, blood component in low-income countries

## Abstract

The outcome of acute myeloid leukaemia (AML) has remained a major concern even in developed countries. In resource poor countries, it is envisaged that the outcome will be far worse because of late presentations, lack of appropriate diagnostic facilities and supportive care. However, data to validate this is lacking and many of these countries lack an effective cancer registry. This study determined the clinician’s perspective of the outcome of care of AML patients in Nigeria and their attitudes to the care of these patients. Structured self-administered questionnaire was used to assess the clinician’s perception of outcomes of care, contributory factors and attitude to care of AML patients. Ninety-eight percent of clinicians reported that the outcome of care was suboptimal; 73.3% and 90.6% of the clinicians reported having less than 31% of AML patients surviving induction and post-induction therapies, respectively. Sixty-six-point one percent (66.1%), 50% and 62.7% of the clinicians have never used immunophenotyping, cytogenetic or molecular studies, respectively, in the management of AML patients under their care. Access to blood components other than Red cells was low; 23.3% had access to apheresis platelets and 55% to fresh frozen plasma. Forty-six percent of clinicians will either give half dose of chemotherapy or offer only supportive care. This reported early death rate is three times higher than that reported in developed countries with only 9% likely to survive the first year of induction compared to about 32.9% in Ontario. Approximately 28 units of pooled or apheresis derived platelet may be required in course of therapy but just 10% of clinicians have access to platelet apheresis. Lack of diagnostic facilities, blood components and clinicians’ attitudes are contributing factors to the extremely poor outcomes of patients with AML in Nigeria.

## Background

Acute myeloid leukaemia (AML) is a heterogeneous group of diseases, characterised by clonal proliferation of immature myeloid cells in the peripheral blood and bone marrow. It is often associated with bone marrow failure.

AML has a global incidence of 351,965 in 2012 with an age-standardised rate of 4·7 per 100,000, a 5-year prevalence of 1.5% and a Male:Female ratio of approximately 1:4 [1]. Its incidence appears to be on the increase in developed countries [2, 3].

The estimated prevalence of AML in Africa is 1% [4]. Africa is expected to witness the highest increase in AML cases over the next 10 years [4]. National prevalence of AML in Nigeria is not available; however, studies report that AML accounts for between 19% and 24% of leukaemias that were managed in hospitals [5–8]. Although these cancers are relatively rare, they have a disproportionally large effect on overall cancer survival statistics. It is the most common type of leukaemia in adults, yet continues to have the lowest survival rate of all leukaemias [9, 10].

The lack of an effective cancer registry, national algorithms for AML reporting and electronic data management systems in Nigeria has made it almost impossible getting population-based data on prevalence and outcomes of patients with AML in the country. Reliable survival statistics are essential for the evaluation of cancer treatments and care.

Moreover, the management of AML in Nigeria is sub-optimal. For example, facilities for cytogenetic and molecular genetics evaluation are not routinely available in Nigeria and samples often must be sent out of the country for patients that can afford the high cost of analysis. Furthermore, blood components are not readily available for the supportive care of the patients. The absence of adequate supportive care specifically, availability of appropriate blood component may also lead to reluctance among haematologists to administer conventional chemotherapy at its full dose, further diminishing the possibility of cure. Likewise, novel drugs such as Midostaurin, Gemtuzumab Ozogamicin, and bone marrow transplant (BMT) facilities offering services to patients with leukaemia are absent. Few Nigerians can afford BMT services outside the country [11]. Arsenic trioxide and all-trans-retinoic acid, drugs used in the treatment of acute promyelocytic leukaemia are also not readily available in the country. Therefore, despite its ease of diagnosis and management, outcome is still poor because drugs must be sourced from outside the country leading to unnecessary delay and even when the drugs are now available, blood component support is still a major challenge. The aforementioned factors taken together mean that delivering best care for AML in Nigeria is impossible.

Aside from a study in 1982 by Williams *et al* [12] which reported that four out of eight children with AML had varying degree of short-lived remission, a systematic literature search of PubMed, Google scholar, African Journals online and African Index online did not reveal any study on survival outcomes of patients with AML in Nigeria. Few studies which reported survival outcomes in haematologic malignancies in Nigeria did not differentiate the leukaemias [13–15]. This absence of data may be a reflection of the weak record system and cancer registry for leukaemia in the nation. Hence, we aimed to determine the clinician’s perspective and attitudes towards management of patients with AML, outcomes of their patients with AML and factors that affect these outcomes to draw attention to the possible sup-optimal level of care in the country.

## Materials and methods

We conducted a comparative cross-sectional study among Nigerian haematologists and haematologists in Training who attended the 44th annual scientific conference of the Nigerian Society of Haematology and Blood Transfusion (NSHBT) held in Calabar, Cross River State, South-South Nigeria from 29 to 31 of August 2018. The respondent must practice and reside in Nigeria to be eligible for recruitment. Membership of NSHBT includes haematologists, haematologists in training, general practitioners, laboratory scientists, nurses and paediatricians who are interested in haematology or blood banking.

All participants at the NSHBT conference who were consultant haematologist or haematologist in training were approached at the conference venue to participate in the study. A structured self-administered questionnaire was given to consenting participants to obtain data. Respondent’s demographic and occupational characteristics including age, sex, religion, place of practice, duration of practice and professional cadre were elicited. Opinion on outcome of care of patients with AML, factors affecting it and attitude of clinicians to the care of patients with AML was assessed using a Likert scale of one to five, which consisted of nine attitude statements. One was strongly disagreed, two was disagree, three was neutral, four agree and five was strongly agree. Positive attitudinal statements were scored five (for participants who strongly agreed) to one (for participants who strongly disagreed). The opposite of this scoring system was used for negative attitudinal statements. Responses were scored in such a way that the higher the score, the more positive the attitude towards care of patients with the leukaemia.

Analysis was conducted using SPSS version 21. Categorical data (such as gender of participants) were presented as frequency, percentages and charts. Continuous data (such as age or attitudinal score of participants) were presented as mean ± standard or median and interquartile range. A reliability analysis of the questionnaire was carried out by comparing the 9-item scale of the clinician’s attitude. Cronbach’s alpha showed the questions to reach acceptable reliability, (*α* = 0.709). Most items appeared to be worthy of retention, resulting in a decreased Cronbach’s alpha if deleted. Item five, however, increased *α* to 0.801 and had a much lower mean, hence it was removed. Responses to negatively structured questions were recoded and attitudinal score was obtained.

Pearson’s Chi square test was used to test for association between attitudes of the physicians and their self-reported survival rate of their patients. Mann–Whitney *U* test was used to assess the relationship between continuous variables and reported survival of AML patients. Confidence interval was set at 95% and the level of statistical significance was set at *p* < 0.05.

### Ethical consideration

Ethical approval was obtained from the Health Research and Ethics Committee of the Lagos University Teaching Hospital (HREC Number: ADM/DCST/HREC/APP/2512) and permission was obtained from the Chairman local organising committee of the 2018 National Society Haematology and Blood transfusion conference. Informed consent was obtained from all participants, confidentiality and anonymity were ensured.

## Results

A total of 65 haematologists/haematologists in training were recruited. More than half of the respondents were consultants (*n* = 39/64, 60%) with a male to female ratio of 1:1.7. The majority of clinicians were from the South-south (*n* = 19, 30.6%) and South-west (*n* = 29, 46.8%) regions of the country. Nearly all (*n* = 59, 90.8%) of the participating clinicians practice at Teaching Hospitals nation-wide. The median year of practice for consultants, senior registrars and junior registrars was 9, 5.5 and 2 years, respectively (Table 1).

Only 1.6%, (*n* = 1/61) of the physicians reported the overall outcome of AML patients under their care to be good, with about 67% of the respondents reporting poor to very poor outcomes. Eight (14.5%) haematologists had between 40% and 50% of patients surviving induction therapy while 2 (3.5%) had 40%–50% surviving post-induction. The majority, 73.2% and 90.6% had less than 31% surviving induction and post-induction, respectively (Table 2).

**Figure 1.**
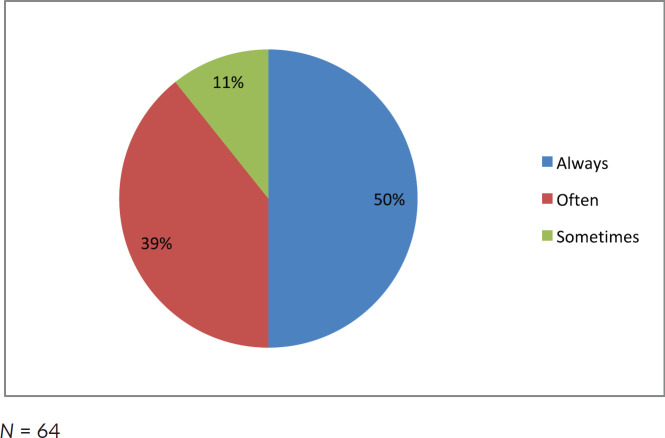
Frequency in which clinicians base the diagnosis of AML solely on morphology.

**Figure 2.**
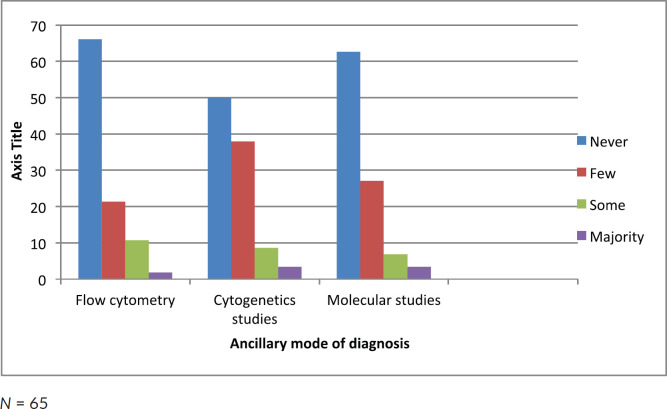
Frequency of ancillary mode of diagnosis used by clinicians in the management of AML.

Half of the participating clinicians diagnosed AML solely on morphology (Figure 1). More than 50% of clinicians in this study did not use any ancillary diagnostic tests such as flow cytometry, cytogenetics and molecular studies (Figure 2).

**Figure 3.**
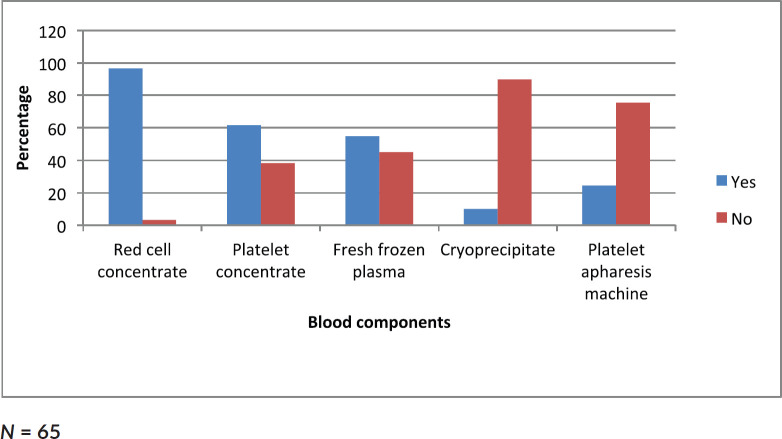
Bar chart showing proportion of clinicians with access to different blood components.

Data collected showed that 96% of clinicians had access to red cell concentrates, 61.7% had access to platelet concentrate, 55% to fresh frozen plasma, 10% to cryoprecipitate and 23.3% to platelet apheresis (Figure 3).

Forty-two (67.7%) out of 62 clinicians were not comfortable managing AML patients and the reasons are outlined below (Table 3). For 26 (62%) of these responders, lack of adequate supportive care was the major reason for not being comfortable managing AML patients.

There was no statistically significant association between the possibility of survival and the professional level of clinicians or the goal of care, place and duration of practice, neither was there a statistically significant association between the possibility of survival and access to blood component support (Table 4). However, a more detailed review of data from the three clinicians that reported five or more patients alive showed that two were from South-West Nigeria, and one was from South-East. The clinician with the highest number of AML patients alive (10) is a haematologist with 30 years of practice, who sees an average of eight AML patients per year, making a total of 180 and a survival rate of 5.5% (10/180). Of the two with five patients alive, one had practiced for 12 years seeing approximately 20 AML patients per year, and the other 9 years with about three AML patients per year. This gives an approximate survival rate of 2.0% and 18.5%, respectively. The clinicians with the highest record of AML patients were from the north of Nigeria (30 patients/year for 19 years of practice; 15 pts/year for 17 years of practice) and neither had any surviving patients.

**Figure 4.**
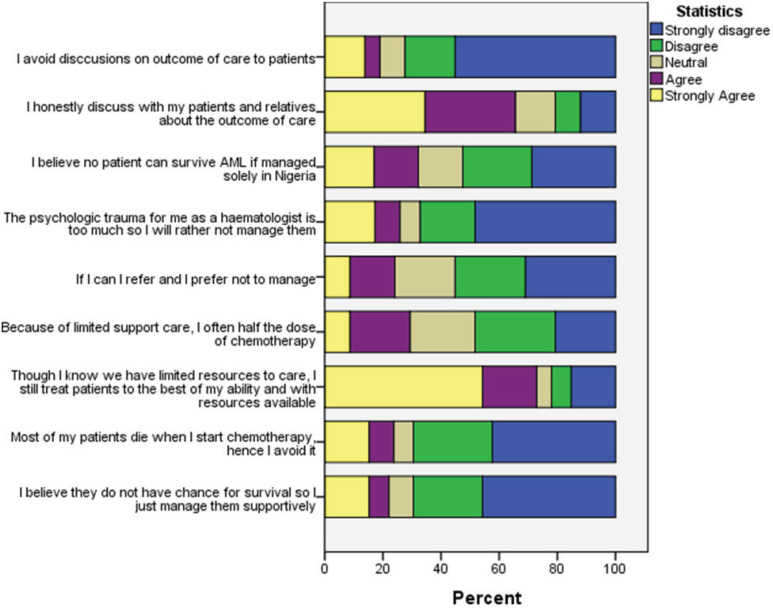
Frequency of response to Likert scale items assessing clinician’s attitude to care of AML patients.

Nineteen (29.2%) clinicians believed that no AML patient could survive if solely treated in Nigeria; 17 (26.2%) of the clinicians will often give half the dose of chemotherapy because of limited supportive care; 14 (21.5%) avoid treatment at all because of the belief that patient will die when chemotherapy is started while 13 (20%) would offer only supportive care, i.e. without chemotherapy because of their perceived chance of survival (Figure 4).

The median attitude score was 2.75 (IQR: 2.375–3.125, range: 1.50–4.13).

### Discussion

This study aimed at using the clinician’s perspective of outcome of AML patients in their care as a surrogate marker for the chance of survival of patients with the leukaemia in Nigeria and to highlight the gross sub-optimal care of AML patients in Nigeria. The attitude of clinicians to the care of patients with AML patients was also evaluated.

Sixty-five haematologists participated in the study, 60% were consultant haematologists and others were residents in training. Median duration of practice for consultants was 9 years while senior registrars were 5 years.

The majority (67%) of the participating clinicians reported that the outcome of patients with AML in Nigeria is poor or very poor. The United States, Surveillance, Epidemiology and End Result Cancer Statistics Review of 1975–2017 showed an improved 5-year survival of patients with AML from 1982 to 2013. In 2013, the overall 5-year survival rate was 27.4% compared to 10.6% in 1986 [16]. Thus, while high income countries are making strides towards reducing the fatality from AML, the situation in Nigeria does not suggest there have been any improvement in care.

We found that about 64% of participating clinicians had <30% of their AML patients surviving remission induction therapy. This can be translated to approximately 70% early death in AML patients in Nigeria. This is far higher than the overall 19% early deaths recorded from the Swedish Acute Leukemia Registry between 1997 and 2005 [17]. The reasons for this is multifactorial and can be broadly divided into patient based and treatment based. Often patients present late having been treated in several health facilities for malaria or ‘typhoid’. Then when they do present, as seen in the attitude of clinicians to care (Figure 4), some would not be commenced on chemotherapy and those who do may have sub-optimal doses and when full doses are given, adequate blood component support is often lacking.

About three-quarters of the participating clinicians reported that less than 30% of AML patients that survived remission induction survived post induction therapy. In order words 70% of the 30% of patients who survived remission induction will die at post-induction chemotherapy. This implies that only 9% of AML patients survive therapy in the first year in contrast to about 32.9% first year survival reported by Alibhai *et al* in a 40-year study on outcomes of care amongst AML patients in Ontario. Thus, it is not surprising that the median number of AML patients said to be surviving by clinicians in Nigeria is zero. The chance of survival for AML patients in Nigeria is grave and there is need for this to be urgently addressed.

Though the clinician with the highest number of AML patients per year (30/year) was from the north, it may not necessarily imply that there are more cases in the north, rather it may reflect the number of haematologist/per population. Table 1 shows that most of the haematologists were from the south. Despite this relatively higher number of patients per year, survival outcomes were worse (0%). The three clinicians who had ≥5 AML patients alive were from the south. This pattern mirrors the availability of blood products. Clinicians from the North West and North East reported they had neither platelet concentrates nor fresh frozen plasma. This may explain the 100% mortality rate (Table 5).

Myelosuppression is a known cause of mortality in AML. Consequently, transfusion support is crucial to the care of patients from the beginning to the end of therapy [18]. However, there is little information on the exact units of transfusion requirements for these patients and the decision on how much to be transfused was solely dependent on the haematologists [19, 20]. Blood components such as platelet concentrates and cryoprecipitate are pooled from several donors. A single platelet dose is derived from approximately four to five donors hence substantial number of donors is needed to provide transfusion requirements to adequately support a single patient through induction and consolidation therapies [19].

A study by Dawson *et al* [19] in 2007 showed that approximately 150 donations were required to pool platelet for patient over the entire course of therapy. Another study carried out in 2015 recoded the median number of red blood cells (RBCs) and platelet transfused during induction therapy to be 12 RBC units (ranging from 0–49) and 10 platelet units (ranging from 0–62) [21]. This is a great challenge in Nigeria as few hospitals have readily available blood component facilities.

Only 10% of clinicians managing AML patients in Nigeria have access to platelet apheresis and almost 38% have no access to platelet concentrates. This is an issue that needs to be addressed to ensure better treatment outcome.

In spite of the advances in molecular diagnosis of AML, morphology of bone marrow cytology remains a diagnostic cornerstone [22, 23]. However, its use as a single diagnostic tool is prone to diagnostic inaccuracies [24]. Some form of AML (for example, AML-M0) cannot be diagnosed based on morphology alone as the blasts cells are large and agranular, resembling L2 cells in acute lymphoblastic leukaemia (ALL) [24]. Ancillary test such as cytochemistry, immunophenotyping, cytogenetic and molecular diagnostics are essential. Another limitation of morphology alone is reproducibility, thereby leading to poor diagnostic concordance. Study has shown that diagnosis of AML using morphology and other ancillary tests achieves an overall concordance rate of approximately 63% [25]. Despite the aforementioned limitation, the French American British morphology classification remains the only step for the diagnosis and assessment of patients with AML in Nigeria. Our study further revealed that 66% of the clinicians had never used flow cytometry in the diagnosis or management of patients with AML, while about half of the respondents never used cytogenetics and 62.7% never used molecular studies. This will not permit for optimal patient care. It also infers that knowledge of these important tests amongst haematologists in Nigeria may be limited.

Diagnostic karyotype and molecular genetics are key determinants in the clinical outcome of AML patients. They help to identify biologically distinct subsets of AML thus allowing for tailored and targeted therapeutic approach. They are also most valuable as prognostic determinant. They have become routine in the characterisation and care of patients with AML. Less than 50% of clinicians in this study use these tools. This will definitely limit the options of care and will negatively impact outcome of care [26].

Studies have shown that in the absence of allogeneic stem cell transplant, adults with AML offered intensive care who are <60 years have only a 16.6% chance of surviving for 10 years [27]. This further buttresses the importance of stem cell transplantation in the management of AML [27]. In this study, the median number of AML patients per clinician who had stem cell transplant was one. Also, ideally risk stratification of AML using the WHO 2016 criteria including cytogenetics and molecular tests described above helps the treating haematologist in determining patients that should be considered for stem cell transplantation.

Aside from performance status and co-morbidity, physician attitude also determines the choice of management of AML [28]. The general attitude of clinicians who take care of patients with AML in this study is Negative (median attitude score: 2.75, interquartile range (IQR) 2.4–3.1). Despite the poor attitude some clinicians, 43 (66.1%) still treat AML patients with optimum care notwithstanding the limitations in the country.

The attitudes of clinicians in the choice of treatment are known to affect the quality of health care rendered and patient’s outcome [29–33]. Study by Hui *et al* [34] showed that oncologists who have favourable attitude towards end of life care of their patients also provide primary palliative care.

This study is limited by recall bias, the inability to evaluate other determinants of survival like age at presentation and the fact that the self-reported values by the respondents are approximation [36, 37]. Despite these, our study still fills a void and provides valuable insight into the outcome of care of patients with AML in Nigeria. Further research should be carried out to assess the outcome of AML in Nigeria.

## Conclusion

In conclusion, outcome of AML treatment in Nigeria is extremely poor. Lack of diagnostic facilities, blood components and clinicians’ attitudes are contributing factors. We recommend improvement in the access to blood components and products via empowering and equipping the national and state transfusion services. We also recommend that the management of leukaemia be included in the National Health Insurance Scheme and anti-leukaemic drugs be added to the National health Insurance scheme medicine list. Finally, we recommend that a well-equipped national leukaemia centre be established where all resources (both diagnostic, therapeutic and clinical trials) for the optimal management of patients with leukaemia could be directed. This will also allow for data on leukaemias in the nation to be collated easily.

## Conflicts of interest

We declare no conflict of interest.

## Funding declaration

This research was self-funded.

## Figures and Tables

**Table 1.** Demographic and clinical experience of respondents.

	Frequency (*n* = 65)	Percentage (%)
**Sex**		
Male	41	63.1
Female	24	36.9
**Marital status**		
Single	8	12.3
Married	55	84.6
Widower	2	3.1
**Religion**		
Christianity	48	73.8
Islam	17	26.2
**Geopolitical zone of practice**		
South south	19	30.6
South west	29	46.8
South central	3	4.8
North central	5	8.1
North east	2	3.2
North west	4	6.5
**Professional level (*n* = 64)**		
Consultant	39	60.0
Senior registrar	12	18.5
Junior registrar	13	20.0
**Place of practice**		
Teaching hospitals	59	90.8
General hospitals	5	7.7
Federal hospitals	1	1.5
**Years of experience in haematology practice (Median (interquartile range)**		
Consultant	9.0 (3.5–17.0)	
Senior registrar	5.0 (3.5–7.0)	
Junior registrar	2.0 (1.25–2.0)	

**Table 2.** Self-reported practice of management of AML by Nigerian physicians.

	Frequency	Percentage %
**Number of AML cases seen per year by clinicians (Median, IQR)**	4 (IQR = 5) years	
**Clinicians who were comfortable with managing AML**	20	32.3
**Perceived outcome of AML patients treated by clinicians**		
Good	1	1.6
Fair	19	31.1
Poor	23	37.7
Very poor	18	29.5
**Proportion of AML patients that survive induction**		
Nil	10	17.9
<10%	15	26.8
11%–20%	12	21.4
21%–30%	4	7.1
31%–40%	7	12.5
40%–50%	8	14.3
Not sure	8	12.5
**Proportion of AML patients that survive post induction**		
Nil	13	24.5
<10%	24	45.3
11%–20%	8	15.1
21%–30%	3	5.7
31%–40%	3	5.7
40%–50%	2	3.8
Not sure	11	17.2
**Has any of your AML patients benefited from BMT^a^**		
Yes	13	22.0
No	46	78.0
**What is your goal of care?**		
Cure	32	54.2
Palliative	27	45.8
**Median number of patients with AML who had a BMT per clinician**	1 (IQR = 1, MIN = 1, MAX = 1)	
**Median no of AML patients alive per clinician**	0	

a BMT, Bone marrow transplant

**Table 3.** Reasons why clinicians were not comfortable managing AML patients.

	Frequency	Percentage %
**High mortality**		
Yes	12	28.6
No	30	71.4
**Late presentation**		
Yes	2	4.8
No	40	95.2
**Lack of diagnostic facilities**		
Yes	6	14.3
No	36	85.7
**Cost/financial constrain**		
Yes	6	14.3
No	36	85.7
**Lack of standard/appropriate drug**		
Yes	8	19.0
No	34	81.0
**Lack of adequate supportive care (blood component therapy)**		
Yes	26	61.9
No	16	38.1

**Table 4.** Relationship between clinician-related and blood component factors and reported possibility of survival for AML patients.

	None alive	Some alive	*p*-value
**Professional cadre**			
Consultant	19 (54.3%)	19 (63.3%)	0.614^a^
Others	16 (45.7%)	11 (36.7%)	
**Goal of care**			
Cure	17 (51.5%)	15 (57.5%)	0.793
Palliative	10 (48.5%)	11 (42.3%)	
**Place of practice**			
Teaching hospital	31(88.6%)	28 (93.3%)	0.275
General hospital	4 (11.4%)	1 (3.3%)	
Federal medical centre	0	1 (3.3%)	
**Duration of practice**			
≤10 years	28 (80.0%)	22 (73.3%)	0.567
>10 years	7 (20.0)	8 (26.7%)	
**Median duration of practice**	4.0 years (2–10)	5.5 years (2–12)	0.895^b^
**Red cell concentrate**			
Yes	32 (97.0%)	27 (96.4%)	0.906
No	1 (3.0%)	1 (3.6%)	
**Platelet concentrate**			
Yes	20 (60.6%)	18 (64.3%)	0.768
No	13 (39.4%)	10 (35.7%)	
**Fresh frozen plasma**			
Yes	16 (48.5%)	17 (60.75)	0.340
No	17 (51.5%)	11 (39.3%)	
**Cryoprecipitate**			
Yes	3 (9.1%)	3 (10.7%)	0.832
No	30 (90.9%)	25 (89.3%)	
**Platelet apheresis machine**			
Yes	7 (20.6%)	8 (28.6%)	0.388
No	27 (79.4%)	19 (67.9%)	
**BMT**			
Yes	4 (11.4%)	5 (18.5%)	0.432
No	31 (88.6%)	22 (81.5%)	

a Fishers exact test

b Mann–Whitney test

**Table 5.** Availability of blood component to clinicians in the six geo-political zones.

	S. East *n* (%)	S. West *n* (%)	S. South *n* (%)	N. Central *n* (%)	N. East *n* (%)	N. West *n* (%)	*p* value
**RBC concentrates**							
Yes	2 (3.4)	29 (50.0)	18 (31.0)	3 (5.2)	2 (3.4)	4 (6.9)	0.382
No	0 (0.0)	0 (0.0)	1 (50.0)	1 (50.0)	0 (0.0)	0 (0.0)	
**Platelet concentrates**							
Yes	2 (5.5)	25 (67.6)	7 (18.9)	3 (8.1)	0 (0.0)	0 (0.0)	<0.001
No	0 (0.0)	4 (17.4)	12 (52.2)	1 (4.3)	2 (8.7)	4 (17.4)	
**Fresh frozen plasma**							
Yes	1 (3.1)	24 (75.0)	4 (12.5)	2 (6.5)	1 (3.1)	0 (0.0)	<0.001
No	1 (3.6)	5 (17.9)	15 (53.6)	2 (7.1)	1 (3.6)	4 (14.3)	
